# Does the interplay between human endogenous retrovirus K and extracellular vesicles contribute to aging?

**DOI:** 10.20517/evcna.2023.45

**Published:** 2023-10-28

**Authors:** Catherine DeMarino, Avindra Nath, Zhengping Zhuang, Tara T. Doucet-O’Hare

**Affiliations:** ^1^Section of Infections of the Nervous System, National Institute of Neurological Disorders and Stroke, Bethesda, MD 20892, USA.; ^2^Neuro-Oncology Branch, National Cancer Institute, Bethesda, MD 20892, USA.; **^#^** Authors contributed equally.

**Keywords:** HML-2, human endogenous retrovirus, aging, senescence, repetitive elements, mobile DNA, transposable elements

## Abstract

The role of extracellular vesicles (EVs), including retroviral-like particles (RVLPs), in pathogenic processes is currently a subject of active investigation. Several studies have identified mechanistic links between the increased presence of EVs and the process of senescence. A recent study reveals that the reverse transcribed complementary DNA (cDNA) of a human endogenous retroviral sequence can activate the innate immune system and result in tissue damage and/or the spread of cellular senescence to distant tissues. Several studies have linked EVs to age-related diseases, such as Alzheimer’s disease and Parkinson’s disease, and have included isolation of EVs from individuals with these diseases. Loss of epigenetic regulation, immune activation, and environmental stimuli can all lead to the expression of endogenous retroviruses and the incorporation of their proteins and transcripts into EVs. In addition, EVs disseminating these endogenous retroviral components have now been shown to act in a paracrine manner in multiple human diseases. Further investigation of the connection between EVs containing endogenous retroviral protein products or nucleotides should be pursued in models of age-related diseases.

## INTRODUCTION

Aging is a process that encompasses physical decline and a higher susceptibility to chronic diseases. The molecular mechanisms underlying it are an area of active scientific inquiry. Recently, there has been a burgeoning interest in exploring the involvement of transposable elements (TEs) in age-related diseases and senescence. Endogenous retroviruses, a type of TE, expand their presence in the genome through a "copy and paste" mechanism involving their transcription, protein expression, and subsequent reverse transcription of their RNA intermediate back into a new location in the genome^[1]^. Several TE classes have been implicated in aging, including retrotransposons both with and without long terminal repeats (LTRs), such as long interspersed elements (LINEs) and endogenous retroviruses (ERVs)^[2-4]^. TEs are passed down in a Mendelian fashion following their integration into the germline and approximately 8% of the human genome is comprised of ERV sequences^[5-8]^. The most recently integrated endogenous retrovirus in the human genome is the Human Endogenous Retrovirus K (HERV-K, sub-type HML-2), which contains multiple open reading frames for viral proteins, such as Gag, Reverse Transcriptase (RT), Envelope (Env), and Protease (Pro)^[7-9]^.

## REGULATION OF ENDOGENOUS RETROVIRUSES

Endogenous retroviral expression is normally tightly spatially and temporally controlled by multiple epigenetic mechanisms, including methylation and heterochromatin facilitated by chromatin remodeling^[10-14]^. There are some endogenous retroviral proteins that play a role in normal physiology, such as the Arc protein, a repurposed retrotransposon Gag gene that mediates the process of intercellular transfer of RNA^[15]^. Early in human development, human endogenous retrovirus promoters are hypomethylated combined with open chromatin, allowing these elements to be expressed; further, their expression appears to be necessary for normal development to proceed^[14]^. In addition, human endogenous retrovirus W produces envelope proteins that are essential for syncytia formation and development of the placenta during pregnancy^[16-18]^. Many Env proteins also contain immunosuppressive domains, which have been co-opted during placental development to protect the developing fetus and can potentially contribute to cancer cells’ evasion of the immune system^[17-20]^. Disruption of envelope expression during placental formation can lead to pathologies caused by disrupted cell fusion and has been observed in cytotrophoblasts, fetuses, and placentas with trisomy 21, gestational diabetes, and preeclampsia^[21-25]^. In early development, even though higher expression of endogenous retroviral elements is critical, it is essential to ensure their proper downregulation as development progresses to prevent pathological consequences. Endogenous retroviruses are downregulated via methylation and chromatin remodeling as development progresses, and the transcription and subsequent translation of their proteins are often observed in pathological conditions, e.g., cancer, neurodegeneration, and most recently, in senescence, the latter of which has recently been associated with age-related disease^[4,10-12,26,27]^.

Endogenous retrovirus transcripts and proteins can be exported in extracellular vesicles (EVs), including retroviral like particles (RVLPs), from cells and operate as a paracrine signaling mechanism which cells use to signal for continued pluripotency and proliferation^[4,10,15,28,29]^. Extracellular vesicles are defined as secreted bilayer lipid membrane-enclosed particles that can derive from nearly any cell type, including neurons, and their role in physiology and pathogenesis is an area of recent scientific interest^[30,31]^. Further, this phenomenon has also been observed in the placenta^[28,32]^. Extracellular vesicles containing endogenous retroviral nucleic acids and proteins have been implicated in the pathogenesis of various conditions, particularly in age-related diseases^[4,27]^. Furthermore, a recent publication revealed the reactivation of endogenous retroviral expression could act as a biomarker and even a driver of aging using multiple cross-species models of premature aging phenotypes^[4]^. In this context, EVs containing HERV-K proteins in one tissue could act via paracrine signaling and result in inflammation and damage in other tissues, akin to the senescence-associated secretory phenotype (SASP) and its role in aging^[33,34]^. This study reveals a role for EVs containing endogenous retroviral proteins and provides evidence that their uptake by recipient cells can lead to aging-associated phenotypes while also showing that these effects can be prevented through therapeutic strategies^[4]^.

**Figure 1:**
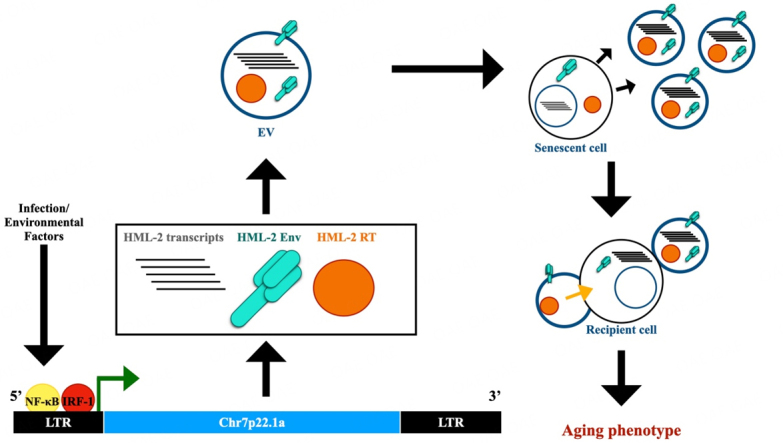
Illustration depicting the expression of an endogenous retrovirus, HERV-K HML-2, and its role in the spreading of a senescent phenotype in vitro. The diagram illustrates HML-2 expression from the Chr7p22.1a locus, which is stimulated by the binding of transcription factors IRF-1 and NF-κB as a result of innate immune activation or environmental factors. HML-2 transcription and translation of HML-2 open reading frames (ORFs) and the subsequent packaging of its transcripts and proteins into an extracellular vesicle are depicted. The extracellular vesicles produced by senescent cells are then able to act in a paracrine manner and can be endocytosed by a recipient cell, resulting in the spreading of an aging or senescent phenotype (as indicated by the yellow arrow).

The aging process occurs over time as cells become senescent and molecular mechanisms that support the process of self-renewal decline. Stingent control of chromatin remodeling and the maintenance of epigenetic mechanisms that control gene expression tend to decrease after an organism reaches its peak reproduction age, allowing the expression of transcripts, such as HML-2, that are typically supressed at earlier time points^[35]^. In addition to epigenetic regulation, several transcription factors associated with the innate immune response, including NF-κB, IRF-1, IRF-3, and IRF-7, have the potential to activate the endogenous retrovirus LTR^[36]^, suggesting that any number of immune stimulants (viruses, bacteria, cell damage derivatives) and environmental factors could contribute to premature aging-related phenotypes[Figure 1]^[37]^. Given the many transcription factor binding sites present in ERV promoters, their activation could contribute to immunosenescence and inflammaging, defined as a state of continued antigenic load and the subsequent stress response that leads to aging, which has been associated with pandemic viruses such as Human Immunodeficiency Virus (HIV) and severe acute respiratory syndrome coronavirus 2 (SARS-CoV-2)^[38-42]^. In fact, ERVs may be a link between infections and the acceleration of aging-related degenerative diseases.

## RECENT ADVANCES

In a recent study, several models of cellular senescence were used to explore a potential role for endogenous retroviral-like particles and EVs containing their proteins in both normal aging and in diseases with premature aging phenotypes, such as Hutchinson-Gilford progeria syndrome (HGPS) and in Werner syndrome (WS)^[4]^. Human mesenchymal progenitor cells (hMPCs) were used throughout the study to establish *in vitro* effects of cellular senescence caused by the expression of HERV-K, a human-specific TE that is normally silenced in mature tissues^[4,11]^. As has been observed in other studies, HERV-K reactivation or "resurrection" is shown to be caused by reduced DNA methylation of its promoters as well as open chromatin in aged cells like those derived from individuals with HGPS or WS^[4,11]^. These models presented a premature aging phenotype, including increased β-galactosidase-positive cells, decreased cellular proliferation, and reduced clonal expansion ability^[4]^. Electron microscopy of wild-type, HGPS, and WS hMPCs in combination with western blotting of their conditioned medium (cm) displayed EVs containing endogenous retroviral proteins that were significantly more highly expressed in HGPS and WS cells.^[4]^ In the same study, using quantitative polymerase chain reactions (qPCR), ribonucleic acid fluorescence *in situ* hybridization (RNA-FISH), and single molecule RNA-FISH (smRNA-FISH), the authors were able to establish that the expression of HERV-K elements in these senescent cells was initiated by transcription at their promoter due to reduced CpG methylation and decreased repressive histone marks. In addition, EVs expressing the HERV-K envelope proteins were observed rarely in early passage hMPCs but were more common in cells with increased markers of senescence, and in primary human fibroblasts isolated from older individuals.

To establish that the senescent phenotypes were directly related to HERV-K expression, a CRISPR-Cas9 activation system was used to upregulate HERV-K element expression in hMPCs and resulted in an increase in senescent features which was reversible with the targeted downregulation of HERV-K transcription^[4]^. Young hMPCs were incubated with EVs isolated from replicative senescent (RS) cells, as well as cells derived from individuals with HGPS and WS, which resulted in decreased proliferation, increased expression of β-galactosidase, and additional senescent phenotypes^[4]^. When the culture medium containing the EVs was immunodepleted with anti-HERV-K antibodies, the senescent effects of the EVs were attenuated on the treated young hMPCs.^[4]^ These data suggested that EVs containing HERV-K transcripts and proteins can act in a paracrine manner and trigger senescent phenotypes in previously non-senescent or young cells [Figure 1]^[4]^.

Multiple HML-2 HERV-K loci in the human genome encode open reading frames for viral proteins such as Gag, RT, Pro, and Env; in addition, elements that encode these proteins are expressed in both normal and diseased tissues^[10,43-45]^. The HERV-K Pol gene encodes a protein that acts as a reverse transcriptase and reverses transcribes RNA into DNA^[46-49]^. As a result of Pol activity, HERV-K DNA increased in the cytoplasm of the senescent hMPCs, which led the authors to investigate whether this DNA could be detected by the DNA sensor cGMP-AMP synthase (cGAS), thereby triggering innate immune system activation^[50-52]^. Using immunoprecipitation, the authors observed increased expression of cGAS in senescent (late passage) hMPCs as compared with early passage hMPCs. Increased phosphorylation of TANK-binding kinase 1 (TBK1), RelA, and interferon (IFN) regulatory factor 3 (IRF3) were also detected, further supporting the activation of the cGAS pathway. Interestingly, both the activation of cGas and senescence-associated phenotypes were abrogated following the treatment of senescent hMPCs with Abacavir, an inhibitor of reverse transcriptase. Taken together, these data emphasize that the expression of a full-length HERV-K provirus is not necessary for significant effects on downstream signaling.

There are only nine HERV-K loci in the human genome that can encode full-length RT; however, only three of these loci also contain functional GAG and Pro ORFs and are likely to result in functional protein [Table 1], and these loci are expressed in some normal tissues^[44]^. When HERV-K RNA or cDNA is packaged into extracellular vesicles, it may avoid detection by the immune system. Extracellular vesicles may be exported from senescent cells, then imported into more distal cells or other tissues and thereby evade the immune system as previously described^[53]^. This phenomenon has been observed for many viruses, including JC polyomavirus, herpes simplex virus 1, hepatitis E virus, hepatitis C virus, and enterovirus 71^[53]^. Therefore, it is critical to understand the role of these extracellular vesicles in a disease context, as they can not only disseminate HERV-K products to distant tissues, potentially in a cell-type specific manner, but they can also simultaneously enable immune evasion.

**Table 1:** HML-2 HERV-K loci with open reading frames for reverse transcriptase

**Locus**	**Chromosomal** **location**	**Strand**	**Type**	**Host gene**	**Expression mode**	**# of Tissues** **expressed**
1q22	Chr1:155626666-155635845	-	1	None	LTR Driven	50
7p22.1a	Chr7:4582426-4591897	-	2	None	LTR Driven	14
11q12.3	Chr11:62368491-62383091	-	2	ASRGL1	Readthrough	2

The Locus name, chromosomal location, strand (sense orientation), HERV-K type, host gene, type of expression, and the number of tissues in which the element is expressed are listed in Table 1. These data were derived from a recent publication in *PLOS Biology*, which analyzed HML-2 HERV-K expression in the normal tissues included in the GTEX cohort version 8 RNA-Seq data^[8,43,44]^. Chromosomal coordinates correspond to Hg38. Expression mode and number of tissues expressed are as reported by Burn, Roy, Freeman, and Coffin, 2022^[44]^. ASRGL1 is Asparaginase and Isoaspartyl Peptidase 1, a protein-coding gene (ENSG00000162174). LTR represents Long Terminal Repeat, the promoter of Human Endogenous Retroviral Elements. Readthrough refers to a transcript whose expression is not driven by the HERV-K promoter but is transcribed due to active transcription near the element^[44]^.

## STUDY LIMITATIONS AND FUTURE DIRECTIONS

To date, no study has determined how the expression of HERV-K contributes to the senescence of brain cells. Further, transcription analysis has not revealed from which loci the HERV-K transcripts originate, which may be key in understanding their activation. Currently, most studies use short-read sequencing, which makes it difficult to predict which loci are producing HERV-K sequences due to the repetitive nature of their sequence. Long-read sequencing should be performed on transcripts isolated from extracellular vesicles produced by senescent cells to determine which HERV-K loci or other transposable elements may play a role in pathology.

Considering the recent developments regarding extracellular vesicles in models of aging, this area should be further investigated in additional advanced aging models such as Alzheimer’s disease (AD) and Parkinson’s disease (PD)^[54]^. In fact, extracellular vesicles have been isolated from individuals with AD, ALS, and PD^[55-57]^. Extracellular vesicles have been isolated from blood in individuals with motor neuron disease and there was a significant increase in the level of the HERV-K envelope protein in individuals with advanced disease compared to those in an earlier phase of the disease^[31]^. In a recent study, when mice were treated with extracellular vesicles isolated from brain lysates and cerebrospinal fluid (CSF) of individuals with PD, they displayed multiple symptoms consistent with PD, such as motor behavior impairment and high anxiety^[56]^. In AD, a significant increase in endogenous retrovirus expression was observed and associated with heterochromatin decondensation and depletion of piwi and piRNAs, which normally act to silence their expression^[58]^. In a recent study, extracellular vesicles containing either HERV-K HML-2 Env or HERV-W Env (Syncytin-1) were shown to increase protein aggregates in vitro, suggesting their expression in diseases like AD could contribute to Tau aggregation^[27]^. These findings suggest that the presence of endogenous retroviral envelope proteins could be used as a biomarker for several age-related neurodegenerative diseases, and this should be further explored in studies with larger numbers of individuals.

Transcripts, proteins, and reverse transcribed cDNAs originating from TEs are often packaged into extracellular vesicles^[10,13,59,60]^. The extracellular vesicles can act as a conduit to spread the TE products throughout the body to other tissues in a paracrine manner and lead to potentially negative consequences in distal tissues. It follows that activation of ERV in one tissue as a result of epigenetic de-repression or stimuli-driven transcription has the potential to impact the entire body despite a tissue-specific origin. In future studies, the contents of extracellular vesicles released *in vitro* from senescent cells should be evaluated with both transcriptomics and proteomics to further characterize which components, in addition to endogenous retroviral transcripts and proteins, could contribute to the phenotype(s) of the recipient cells. Further, liposomes containing only endogenous retroviral proteins and transcripts should be tested in animal models to determine whether they are sufficient to cause a senescent phenotype *in vivo*^[61]^.

## CONCLUSION

We and others have shown that loss of function of chromatin remodeling proteins in developmental tumors, e.g., Atypical Teratoid Rhabdoid Tumor (AT/RT), leads to the expression of endogenous retroviral elements^[10,62]^. AT/RT has a bimodal distribution of disease in both young and older individuals, highlighting the possibility of endogenous retroviral involvement in tumors occurring later in life^[62]^. A key epigenetic regulator, the chromatin remodeling SWItch/Sucrose Non-Fermentable (SWI/SNF) complex, plays an important role in the transcriptional activation of stress-response genes that are often activated during aging^[63]^. In addition, the expression of endogenous retroviruses is tightly spatially and temporally regulated during development, and they are normally downregulated as cells differentiate and mature^[11,64]^. Therefore, endogenous retroviral expression may be an excellent treatment target for neurodevelopmental tumors, neurodegenerative diseases, and senescence, and current treatments, e.g., anti-retroviral drugs and ERV targeting short hairpin RNAs (shRNAs), designed to abrogate their expression should potentially be explored^[10,26,65]^. Recent work in the field, the plethora of tumors with chromatin remodeling defects and ERV involvement, and the noted role of ERVs in other age-related diseases suggest further investigation is needed to understand the interplay between extracellular vesicles, endogenous retroviruses, and aging-related pathologies.

## DECLARATIONS

### Copyright

© The Author(s) 2023.
